# Subtherapeutic Dose of Ionizing Radiation Reprograms the Pre-Metastatic Lung Niche, Accelerating Its Formation and Promoting Metastasis

**DOI:** 10.3390/ijms26136145

**Published:** 2025-06-26

**Authors:** Paula de Oliveira, Inês Sofia Vala, Pedro Faísca, Joao C Guimaraes, Filomena Pina, Esmeralda Poli, Isabel Diegues, Hugo Osório, Rune Matthiesen, Karine Serre, Susana Constantino Rosa Santos

**Affiliations:** 1 Centro Cardiovascular da Universidade de Lisboa (CCUL@RISE), Faculdade de Medicina, Universidade de Lisboa, 1649-028 Lisboa, Portugal; paulaoliveira@medicine.ulisboa.pt (P.d.O.); ines.oliveira@medicina.ulisboa.pt (I.S.V.); 2 Instituto Gulbenkian de Ciência, 2780-156 Oeiras, Portugal; pedro.faisca@gimm.pt; 3 Faculdade de Medicina, Universidade de Lisboa, 1649-028 Lisboa, Portugal; joao.guimaraes@medicina.ulisboa.pt; 4 Santa Maria Hospital, Unidade Local de Saúde de Santa Maria (ULSSM), 1649-035 Lisboa, Portugal; filomena.pina@ulssm.min-saude.pt (F.P.); esmeralda.poli@ulssm.min-saude.pt (E.P.); isabel.diegues@ulssm.min-saude.pt (I.D.); 5 i3S—Instituto de Investigação e Inovação em Saúde, Universidade do Porto, 4099-002 Porto, Portugal; hosorio@i3s.up.pt; 6 IPATIMUP—Instituto de Patologia e Imunologia Molecular, Universidade do Porto, 4099-002 Porto, Portugal; 7 FMUP—Faculdade de Medicina, Universidade do Porto, 4099-002 Porto, Portugal; 8 Computational and Experimental Biology Group, iNOVA4Health, NOVA Medical School, Faculdade de Ciências Médicas, Universidade NOVA de Lisboa, 1349-008 Lisboa, Portugal; rune.matthiesen@nms.unl.pt; 9 Instituto de Medicina Molecular João Lobo Antunes, Faculdade de Medicina, Universidade de Lisboa, 1649-028 Lisboa, Portugal; karine.serre@gimm.pt

**Keywords:** pre-metastatic niche, subtherapeutic dose of ionizing radiation, metastasis

## Abstract

Pre-metastatic niche (PMN) formation is a critical step in metastatic progression. However, the biological effects of subtherapeutic doses of ionizing radiation (SDIRs) following radiotherapy on this process remain unclear. Using a 4T1 breast cancer mouse model, we investigated the effects of SDIRs (3 × 0.3 Gy) on lung PMN development and metastasis upon SDIR exposure on days 8–10 post-tumor injection, followed by mastectomy and analyzed on day 24. SDIRs significantly increased the total metastatic volume (TMV) in lungs, suggesting an accelerated PMN formation. Mechanistically, the SDIR acted as an early catalyst for niche priming, upregulating *Bv8* expression, enhancing neutrophil recruitment, and increasing MMP9, S100A8, and *Il6* production in the PMN by day 11. Moreover, SDIR drives metastasis through distinct mechanisms. Proteomic analysis revealed SDIR-driven metabolic reprogramming, with a shift away from fatty acid metabolism toward glycolysis and lipid accumulation within the PMN. This shift contributes to extracellular matrix (ECM) remodeling, immune modulation, and the upregulation of adhesion-related pathways, shaping a microenvironment that accelerates metastatic outgrowth. By reprogramming the pre-metastatic lung, the SDIR highlights the need to integrate organ-specific radiation exposure into metastasis models. Metabolic and immune-stromal pathways emerge as potential therapeutic targets, underscoring the importance of refining radiotherapy strategies to mitigate unintended pro-metastatic effects.

## 1. Introduction

Radiotherapy is a cornerstone of cancer treatment, typically delivered in fractionated schemes in which the tumor receives daily doses over several weeks to achieve a therapeutic effect [1]. While the primary goal is to eradicate malignant cells, healthy tissues surrounding the treatment field are inevitably exposed to varying levels of ionizing radiation, determined not only by their anatomical proximity to the targeted area but also by the inherent limitations of the radiation delivery method. Consequently, secondary organs are exposed to cumulative radiation doses that vary in magnitude and impact. While moderate-to-high doses are associated with adverse effects such as radiation-induced cardiotoxicity and pneumonitis, particularly in breast cancer patients [2,3,4], lower doses, though not overtly toxic, can still induce biological changes over time [4,5,6,7,8,9].

Subtherapeutic doses, typically below 1 Gy, are encountered in several clinically relevant scenarios, including scatter radiation to adjacent healthy tissues during external beam radiotherapy and cumulative exposure from repeated diagnostic imaging procedures. Moreover, environmental or occupational exposure in medical or nuclear industries can also result in comparable low-dose radiation accumulation. These contexts highlight the clinical significance of SDIRs, particularly in patients undergoing repeated radiation treatments or those with tumors located near radiosensitive organs.

Recent studies indicate that, within the subtherapeutic range, doses below 0.8 Gy can phosphorylate VEGFR2, modulating signaling pathways and gene expression, ultimately leading to endothelial cell activation and increased microvascular density [8,9]. Interestingly, the SDIR has been shown to accelerate angiogenic sprouting during embryonic development and enhance inter-ray vessel density following caudal fin amputation and regeneration in adult zebrafish, further supporting its pro-angiogenic effects across different biological contexts [6,9]. These findings gain further relevance as low doses have also been shown to activate endothelial cells in human tissues [5]. In patients with locally advanced rectal cancer undergoing neoadjuvant radiotherapy, paired biopsies revealed increased microvasculature density and the upregulation of angiogenic genes in endothelial cells exposed to low doses compared to internal calibrator tissues [5]. These data strongly suggest that SDIR-induced vascular remodeling is not merely an experimental observation but a clinically relevant process. Moreover, following primary tumor induction, whole-body irradiation markedly increases metastases in the predicted target organ [9,10], suggesting that the SDIR plays a role in PMN modulation. The PMN is a dynamic microenvironment within distant organs that is primed to facilitate the colonization of circulating tumor cells before metastatic dissemination occurs [11,12,13]. Its formation is orchestrated by a complex interplay of molecular and cellular events, including stromal remodeling, endothelial activation, immune modulation, and ECM restructuring, collectively creating a permissive landscape for tumor cell seeding and outgrowth. A key hallmark of PMN formation is the recruitment of neutrophils, macrophages, and bone marrow-derived suppressor cells, guided by systemic signals that precondition distant organs for tumor cell arrival [11,13,14,15,16,17,18,19,20]. Among these, Bv8 plays a pivotal role in neutrophil mobilization to pre-metastatic sites, fostering an inflammatory microenvironment that supports metastatic progression [21,22,23]. Additionally, inflammatory mediators and endothelial activation induce the upregulation of proteins such as S100A8 and MMP9, which promote ECM remodeling and increase vascular permeability, further facilitating metastatic cell adhesion and extravasation [17,21,22,24]. IL-6, a pro-inflammatory cytokine elevated in response to SDIRs, reinforces immune suppression and enhances angiogenesis, amplifying the niche’s capacity to sustain disseminated tumor cells [25,26,27]. Collectively, these alterations transform distant organs into a permissive “soil” for metastatic colonization, underscoring the intricate molecular landscape that precedes overt metastasis. Given the emerging evidence that the SDIR profoundly reshapes vascular and immune dynamics in distant organs, elucidating its impact on PMN reprogramming is essential. Whether this modulation fosters or restrains metastatic colonization remains an open question with critical implications for understanding the broader effects of radiation exposure on metastasis. Here, we investigate how the SDIR shapes the PMN, offering new insights into its role in metastatic initiation and progression. Within the SDIR range, the PMN was exposed to 0.3 Gy, previously described as both pro-angiogenic and anti-inflammatory, administered over three consecutive days. This fractionated schedule was designed to mimic clinical radiotherapy while targeting the PMN during a temporal window in which it remains uncolonized by tumor cells, thereby allowing us to evaluate the specific effects of radiation on niche reprogramming without interference from direct tumor cell interactions.

## 2. Results

### 2.1. SDIR Promotes Lung Metastasis in a Mouse Model of Breast Cancer

**Figure 1:**
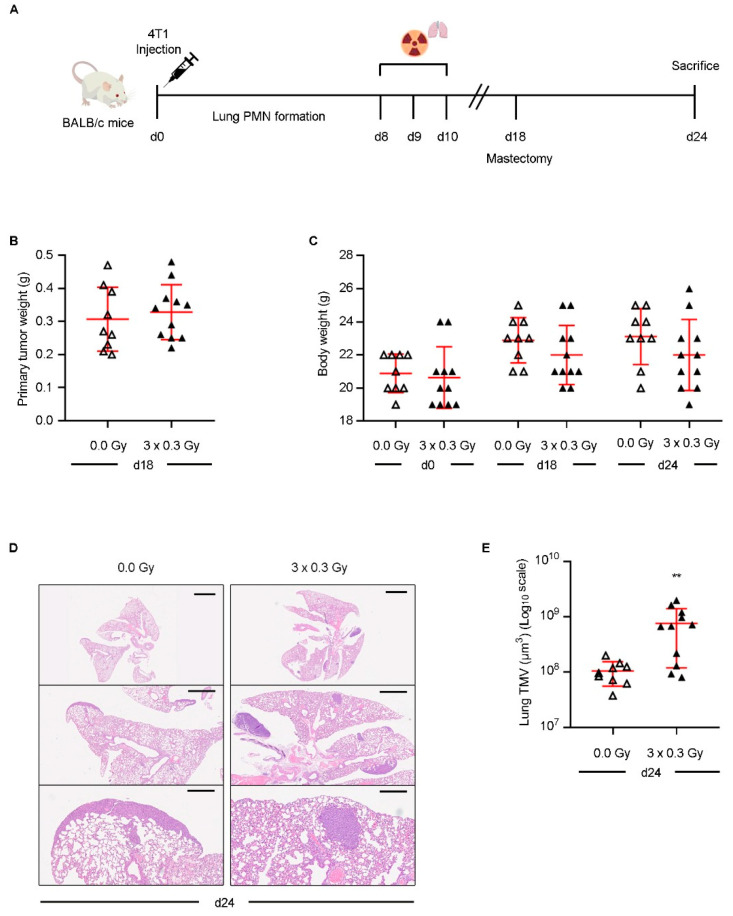
**Experimental design and effect of SDIR on tumor progression and lung metastasis in a 4T1 breast cancer mouse model:** (**A**) Experimental design: The fourth mammary gland of Balb/c mice is injected subcutaneously on day 0 (d0) with 4T1 tumor cells. PMN development followed tumor cell injection. The PMN is either sham-irradiated (0.0 Gy) or exposed to 0.3 Gy for 3 consecutive days (3 × 0.3 Gy) on days 8, 9, and 10 (d8–d10). Mastectomy is performed on day 18 (d18), and the experimental endpoint is reached on day 24 (d24). (**B**) On day 18 (d18, immediately after mastectomy), the primary tumor weight is recorded. (**C**) Body weight is measured in both experimental groups on days 0, 18, and 24 (d0, d18, d24). (**D**,**E**) On day 24 (d24), lungs are collected for stereological analysis. (**D**) Representative hematoxylin and eosin-stained histological sections from the sham-irradiated (0.0 Gy) and 3 × 0.3 Gy experimental groups are shown. (**E**) Quantitative analysis of the TMV in the sham-irradiated (0.0 Gy) versus the 3 × 0.3 Gy on day 24 (d24) is shown. Scale bar: 2.5 mm (upper panels), 1.0 mm (middle panels), 250 µm (lower panels). (**B**–**D**) Individual data and mean ± SD (in red) are shown; n = 9–11. Data are assumed to follow a normal distribution, and an independent two-tailed *t*-test with Welch’s correction for unequal variances is applied. Statistical significance: *p* < 0.01 (**).

To investigate the effect of SDIRs on lung PMN formation and subsequent metastatic progression, we used a well-established animal model of spontaneous lung metastases. In this model, 4T1 breast cancer cells were injected into the fourth mammary fat pad of female BALB/c mice (day 0), allowing primary tumor development and sequential establishment of lung PMN. To define a temporal window for SDIR exposure while ensuring the absence of tumor colonization, a histological analysis of lung specimens was performed on days 10 and 20 post-primary tumor induction. No tumor cells were detected on day 10, whereas by day 20, 83% of animals exhibited lung metastasis (Figure S1). Based on these findings, the thoracic area of mice was exposed to SDIRs on days 8, 9, and 10 post-tumor injection. The primary tumor was surgically resected on day 18, and mice were monitored until the experimental endpoint on day 24 (Figure 1A). Consistent with the fact that only the thoracic region was irradiated, primary tumor weights were comparable across all experimental groups on day 18 (Figure 1B), and no differences in body weight were observed throughout this study (Figure 1C). Lung metastases were confirmed by pathological examination, as illustrated by representative histological images (Figure 1D). Notably, lung stereological analysis revealed a significant increase in TMV in the SDIR-exposed PMN group compared to the sham-irradiated group (Figure 1E).

### 2.2. The SDIR Accelerates the Development of a Permissive Microenvironment by Increasing the Expression of Bv8, Neutrophil Recruitment, and the Production of MMP9, S100A8, and Il6 in the Lung PMN

**Figure 2:**
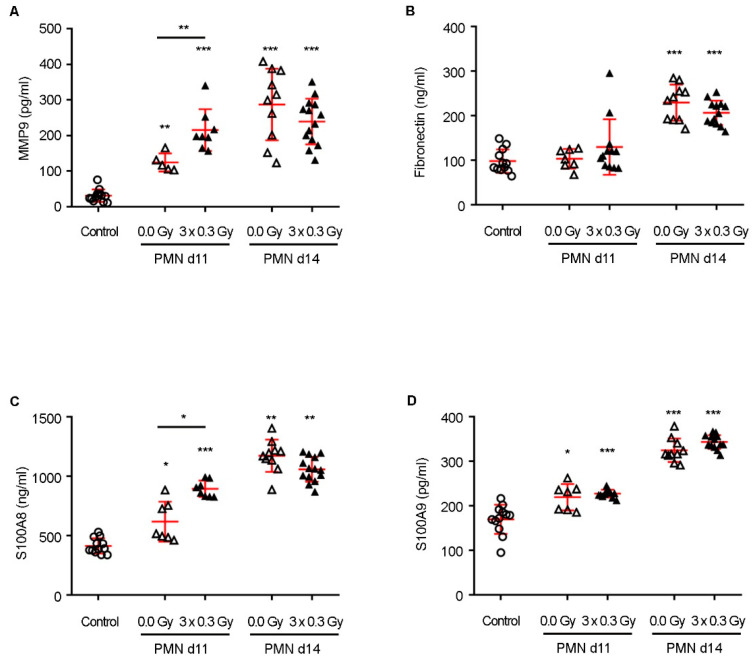
**Effect of SDIRs on the PMN lung microenvironment and expression of metastatic niche-associated factors in a 4T1 breast cancer mouse model.** Following 4T1 tumor cell injection, a PMN is established. The PMN is then either sham-irradiated (0.0 Gy) or exposed to 0.3 Gy for 3 consecutive days (3 × 0.3 Gy) on days 8, 9, and 10 (d8–d10). An additional control group is included, consisting of lungs from non-tumor-bearing, non-irradiated mice (control). Lung tissue samples are collected on days 11 or 14 (d11 or d14) and analyzed by ELISA. (**A**) MMP9, (**B**) fibronectin, (**C**) S100A8, and (**D**) S100A9 are quantified in both PMN experimental groups when compared to the control. (**A**–**D**) Individual data and mean ± SD (in red) are shown; n = 5–14. Values assumed normal distribution. For days 11 and 14 (d11 and d14), between-group changes are assessed by Welch’s ANOVA followed by a Tamhane T2 post hoc test to account for unequal sample sizes in the groups and unequal variance. Statistical significance: *p* < 0.05 (*), *p* < 0.01 (**), and *p* < 0.001 (***).

To assess the impact of SDIRs on lung PMN formation, we quantified key factors involved in its modulation, known to create a permissive microenvironment for metastatic seeding. Given their established roles in pre-metastatic remodeling, PMN lung tissue samples from days 11 and 14 post-tumor induction were analyzed for MMP9, fibronectin, and the chemoattractant cytokines S100A8 and S100A9 using ELISA assays. MMP9 facilitates ECM degradation, enhancing tumor cell migration, whereas fibronectin supports metastatic cell adhesion, and S100A8/S100A9 contribute to myeloid cell recruitment, generating a pro-inflammatory environment niche [17,21,22]. As expected, PMN lung tissue exhibited significantly higher expression of these factors compared to control lungs from non-tumor-bearing, non-irradiated mice (Figure 2A–D).

Remarkably, the SDIR further potentiated these alterations, significantly increasing MMP9 and S100A8 expression in the PMN on day 11 compared to non-irradiated PMN (Figure 2A,C). By day 14, however, the expression of all assessed markers had markedly increased in both PMN groups, with no significant differences between SDIR-exposed and non-irradiated conditions (Figure 2A–D), indicating that the SDIR primarily functions as an early driver of PMN priming.

**Figure 3:**
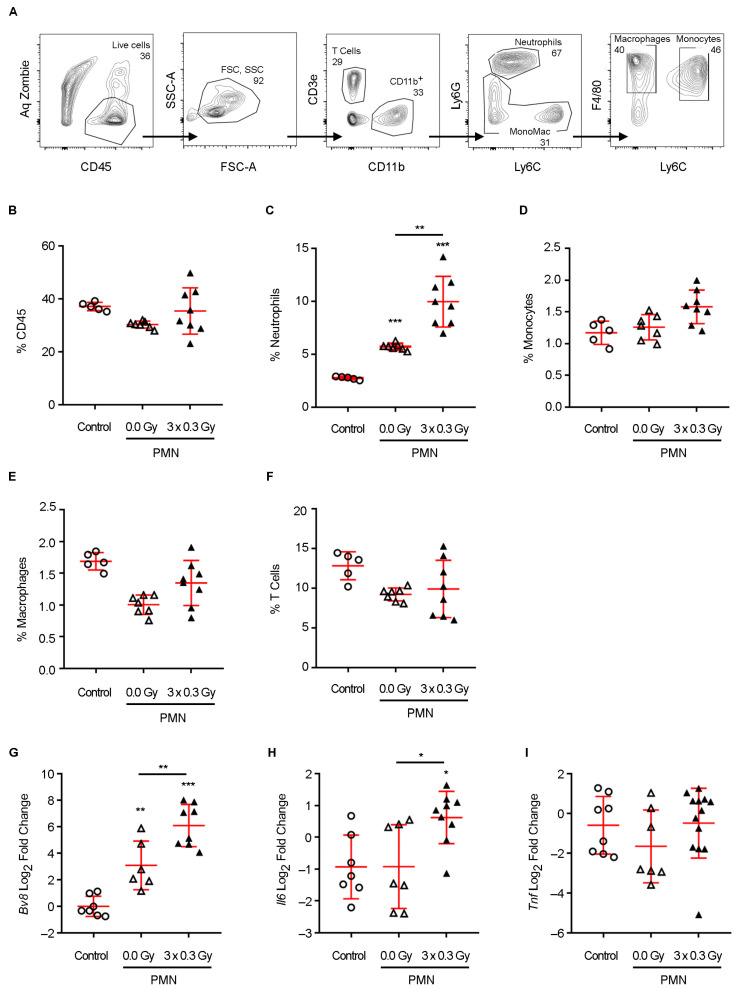
**Impact of the SDIR on immune cell populations and pro-inflammatory gene expression in the lung PMN of a 4T1 breast cancer mouse model.** Following 4T1 tumor cell injection, a PMN is established. The PMN is then either sham-irradiated (0.0 Gy) or exposed to 0.3 Gy for 3 consecutive days (3 × 0.3 Gy) on days 8, 9, and 10 (d8–d10). An additional control group is included, consisting of lungs from non-tumor-bearing, non-irradiated mice (control). Lung tissue samples are collected on day 11 and analyzed by flow cytometry (**A**–**F**) and qRT-PCR (**G**–**I**). (**A**) Representative analysis of the hematopoietic CD45+ cells, including T cells (CD45+CD11b-CD3+), neutrophils (CD45+CD11b+Ly6G+ cells), macrophages (CD45+CD11b+F4/80+ cells), and monocytes (CD45+CD11b+LY6C+F4/80int cells), are presented in the lung tissue. The percentage of (**B**) total CD45+, (**C**) neutrophils, (**D**) monocytes, (**E**) macrophages, and (**F**) T cells and the (**G**–**I**) *Bv8*, *Il6,* and *Tnf* mRNA expression are shown. Between-group changes are assessed by one-way ANOVA followed by a Bonferroni post hoc test (**D**,**E**,**G**,**H**) or Welch’s ANOVA with Tamhane T2 post hoc test when unequal variance is verified (**B**,**C**,**F**). When values do not follow a normal distribution (**I**), Kruskal–Wallis with Dunn’s correction is used. Statistical significance: *p* < 0.05 (*), *p* < 0.01 (**), and *p* < 0.001 (***).

Given the role of neutrophils as a primary source of MMP9 and S100A8 in the PMN [28], their recruitment was assessed by flow cytometry (Figure 3A). Neutrophil infiltration was significantly increased in both PMN groups compared to the control (Figure 3C), whereas other immune cell populations (CD45+ cells, monocytes, macrophages, and T cells) remained unchanged (Figure 3B,D–F). Notably, SDIRs further increased neutrophil recruitment to the PMN compared to non-irradiated conditions (Figure 3C). Since Bv8 promotes neutrophil mobilization and their homing to the PMN [17,21], we next analyzed its expression. The SDIR-exposed PMN exhibited significantly higher *Bv8* mRNA expression on day 11 compared to non-irradiated conditions (Figure 3G). Given that neutrophils contribute to tumor cell extravasation and proliferation through *Bv8*, MMP9, and S100A8 but also release potent inflammatory mediators upon activation, the mRNA expression levels of *Il6* and *Tnf* were also assessed in the PMN on day 11 [25,27]. While SDIRs significantly increased *Il6* expression in the PMN (Figure 3H), no differences were observed for *Tnf* (Figure 3I).

**Figure 4:**
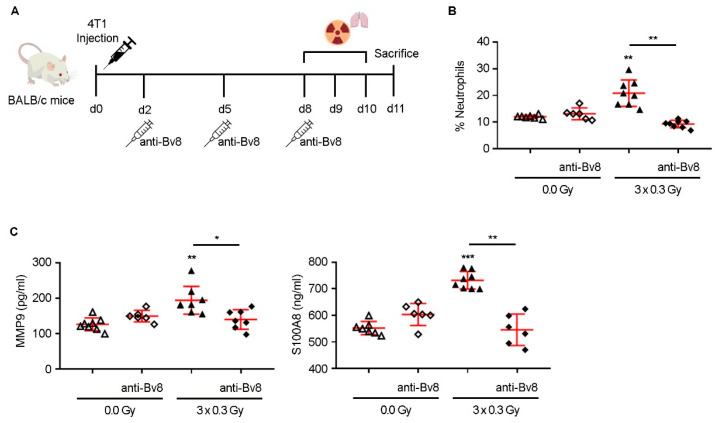
**Experimental design and effect of anti-Bv8 treatment and SDIR on neutrophil recruitment and lung PMN-associated factors in a 4T1 breast cancer mouse model:** (**A**) Experimental design: The fourth mammary gland of Balb/c mice is injected subcutaneously on day 0 (d0) with 4T1 tumor cells. After tumor cell injection, lung PMN is developed. Anti-Bv8 treatment is administered or not intraperitoneally on days 2, 5, and 8 (d2, d5, d8). The PMN is either sham-irradiated (0.0 Gy) or exposed to 0.3 Gy for 3 consecutive days (3 × 0.3 Gy) on days 8, 9, and 10 (d8–d10). On day 11 (d11) mice lung tissue samples are collected. (**B**) The percentages of neutrophils determined by flow cytometry are shown. (**C**) MMP9 (**left panel**) and S100A8 (**right panel**) are quantified by ELISA. Statistical significance is determined using Welch’s ANOVA followed by Tamhane’s T2 post hoc test (**B**,**C**). Statistical significance: *p* < 0.05 (*), *p* < 0.01 (**) and *p* < 0.001 (***).

To determine whether SDIR-induced neutrophil recruitment was mediated by Bv8, mice were treated with anti-Bv8 monoclonal antibodies (mAbs) before irradiation, as illustrated in Figure 4A. Anti-Bv8 completely abrogated SDIR-induced neutrophil recruitment (Figure 4B), with no significant changes in other immune populations, apart from a slight decrease in CD45+ cells, likely reflecting the specific impact on neutrophils (Figure S2). Consistently, the SDIR-induced increase in MMP9 and S100A8 expression was also abolished by anti-Bv8 treatment, reverting to levels observed in non-irradiated PMN (Figure 4C).

Taken together, these findings indicate that SDIR accelerates PMN formation by upregulating Bv8 expression, enhancing neutrophil recruitment, and increasing MMP9, S100A8 and Il6 production. However, by day 14, while S100A8 and MMP9 expression remained elevated compared to controls, no differences were observed between irradiated and non-irradiated conditions (Figure 2A,C), suggesting the involvement of additional mechanisms in SDIR-driven metastatic progression. Notably, blocking SDIR-induced neutrophil recruitment failed to prevent the significant increase in TMV (Supplementary Figure S3), highlighting that other factors contribute to this process. This was demonstrated by assessing the impact of neutrophil depletion on metastatic outgrowth. Mice were treated with anti-Bv8 mAbs on days 2, 5, 8, and 11 post-tumor injection while exposed to SDIR between days 8 and 11. Primary tumors were resected on day 18, and mice were monitored until day 24, when they were sacrificed for lung stereological analysis (Supplementary Figure S3A). As expected, primary tumor weight and body weight remained comparable across all experimental groups throughout the study (Supplementary Figure S3B,C). However, lung stereological analysis showed that TMV remained significantly in-creased in the SDIR group, with no significant differences between anti-Bv8-treated and untreated mice (Supplementary Figure S3D). Representative histological images are provided in Supplementary Figure S3E.

### 2.3. Proteomic Profiling of PMN in Response to SDIR Reveals Metabolic Reprogramming, ECM Remodeling, and Upregulation of Adhesion-Related Pathways

Given the impact of SDIRs on TMV and PMN formation, we conducted a proteomic analysis of the PMN on day 11 using mass spectrometry (MS) to investigate the molecular alterations driving this process.

Four to five biological replicates were collected from the following experimental conditions: the SDIR-exposed PMN, non-irradiated PMN, and control lung tissue from non-tumor-bearing, non-irradiated mice. Briefly, lung tissue from each experimental group was collected and lysed, followed by cysteine alkylation and enzymatic digestion of the extracted proteins with trypsin into peptides. Protein identification and quantification were achieved using high-resolution MS, yielding a median coefficient of variation below 3.9%, indicating high reproducibility across all conditions. The overall distribution of quantitative data was comparable among samples (Figure S4).

**Figure 5:**
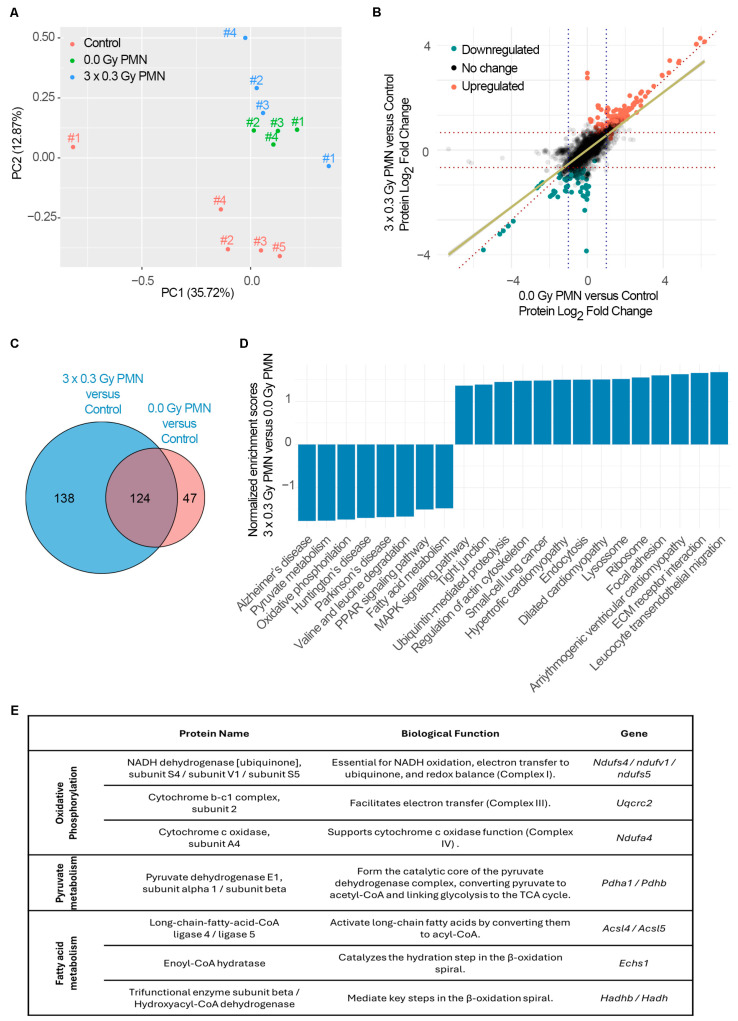
**Proteomic analysis of the lung PMN following SDIR exposure in a 4T1 breast cancer mouse model.** After 4T1 tumor cell injection, a PMN is established. The PMN is then either sham-irradiated (0.0 Gy) or exposed to 0.3 Gy for 3 consecutive days (3 × 0.3 Gy) on days 8, 9, and 10 (d8–d10). An additional control group is included, consisting of lungs from non-tumor-bearing, non-irradiated mice (control). Lung tissue samples collected on day 11 are analyzed by mass spectrometry-based proteomics and GSEA. (**A**) Principal component (PC) analysis of proteomic profiles showing distinct clustering between control (red) and PMN (green and blue), with subtle differences between the SDIR-exposed (blue) and sham-irradiated (green) PMN. (**B**) Differential protein expression analysis comparing 3 × 0.3 Gy PMN vs. control and 0.0 Gy PMN vs. control, highlighting upregulated (red) and downregulated (green) proteins in the 3 × 0.3 Gy PMN vs. control comparison. A strong correlation is shown between SDIR-exposed and sham-irradiated PMN proteomes (Pearson r = 0.78, *p* < 2.2 × 10^−16^). (**C**) A Venn diagram illustrates the overlap of differentially expressed proteins in 3 × 0.3 Gy of PMN (blue) and 0.0 Gy of PMN (red), with a subset of proteins uniquely modulated by the SDIR. (**D**) GSEA comparing SDIR-exposed (3 × 0.3 Gy) and sham-irradiated PMN (0.0 Gy), showing the most significantly down- and upregulated KEGG pathways. (**E**) Key differentially expressed proteins that contribute most to the identified KEGG metabolic pathways. Proteins are categorized based on their involvement in oxidative phosphorylation, pyruvate metabolism, and fatty acid metabolism, with their corresponding biological functions and gene names indicated.

Principal component analysis revealed a distinct separation between control lung tissue and PMN samples, with subtle differences observed between the SDIR-exposed and non-irradiated PMN (Figure 5A). Differential expression analysis between the PMN and control lungs showed consistent fold changes in the PMN exposed to SDIRs and the non-irradiated PMN (Pearson correlation coefficient of 0.78, *p*-value < 2.2 × 10^−16^) (Figure 5B). The Limma package with multiple testing correction was used to identify 309 differentially expressed proteins in the lung PMN (Figure 5C), confirming the establishment of a PMN by day 11.

Venn diagrams were used to assess the overlap of significantly dysregulated proteins. Interestingly, of the 262 proteins observed in the PMN exposed to SDIRs, 138 were exclusively modulated by SDIRs, while 124 were found in both the SDIR-exposed and non-irradiated PMN (Figure 5C). As expected, among these shared proteins, S100A8 and MMP9 were identified as key proteins (Figure S5).

Gene set enrichment analysis (GSEA) further revealed significant changes in the transcriptomic response of the PMN following SDIR exposure. Pathways associated with increased neutrophil activity (GO: BP Myeloid Leukocyte Mediated Immunity, Normalized Enrichment Score (NES) of 2.12, False Discovery Rate (FDR) < 0.0001) and enhanced ribosomal RNA biogenesis (GO: BP rRNA Metabolic Process, NES = 1.41, FDR < 0.01) were upregulated in the SDIR-exposed PMN compared to controls. In addition, the KEGG pathway for Leukocyte Transendothelial Migration (NES = 1.67, FDR = 0.12) was upregulated in the irradiated PMN, suggesting that the increased neutrophil abundance may result from more efficient migration across the vascular endothelium.

To identify the protein dysregulation differences contributing to the observed increase in TMV (Figure 1D), the SDIR-exposed PMN was compared with the non-irradiated PMN. GSEA revealed a marked decrease in pyruvate metabolism, a reduction in the degradation of valine, leucine, and isoleucine, and a significant downregulation of fatty acid metabolism in the SDIR-exposed PMN (Figure 5D). These changes suggest a metabolic shift from oxidative phosphorylation towards a glycolytic process, characteristic of a Warburg-like phenotype, commonly observed in early pre-metastatic stages [29]. This shift leads to increased lactate production, even under normoxic conditions, resulting in the acidification of the microenvironment, which may contribute to immune suppression and create a favorable condition for tumor cell colonization [29,30].

Moreover, this metabolic reprogramming reduces reliance on mitochondrial oxidative phosphorylation for ATP production, redirecting cellular resources toward the synthesis of metabolites that support tissue remodeling, such as amino acids and lipids [31,32]. This reprogramming may enhance the PMN’s ability to promote cell migration and invasion, as evidenced by the upregulation of proteins involved in ECM remodeling (e.g., MMP9) and those associated with cell adhesion and migration, including components of focal adhesion complexes and actin cytoskeleton rearrangement [29]. Importantly, the SDIR-exposed PMN exhibited an increased expression of genes related to ECM interactions, cell junctions, and adhesion (Figure 5D), further corroborating our findings.

Additionally, we identified key differentially expressed proteins that primarily contribute to the previously identified KEGG pathway, as determined by core enrichment analysis (rank metric score), and play a crucial role in metabolic reprogramming. Proteins essential for mitochondrial oxidative phosphorylation, including NDUFS4, NDUFV1, NDUFS5, UQCRC2, and NDUFA4, were significantly downregulated. These proteins are involved in facilitating electron transfer across complexes I, III, and IV, thereby maintaining cellular energy production and redox balance [33]. Similarly, PDHA1 and PDHB, which form the catalytic core of the pyruvate dehydrogenase complex linking glycolysis to the TCA cycle [32], were downregulated, along with ACSL4/ACSL5, ECHS1, and HADHB/HADH, which play a role in fatty acid breakdown for energy production [31] and were also downregulated. These proteins are integral to oxidative phosphorylation, pyruvate metabolism, and fatty acid metabolism, and they are categorized accordingly in Figure 5E.

Taken together, our data demonstrate SDIR-induced metabolic reprogramming, ECM remodeling, and cell adhesion in the lung PMN, establishing a favorable microenvironment for metastatic progression.

## 3. Discussion

Neutrophil recruitment plays a pivotal role in shaping the PMN, acting as a critical intermediary between systemic inflammatory signals and metastatic progression. Elevated Bv8 expression has been identified as a key driver of neutrophil mobilization to pre-metastatic organs, where these cells contribute to niche conditioning by secreting a repertoire of pro-metastatic mediators [22,34]. Among these, MMP9 facilitates ECM degradation, enhancing vascular permeability and promoting tumor cell extravasation, while S100A8 fosters a pro-inflammatory microenvironment that sustains neutrophil survival and function [17,22,34]. Additionally, IL-6, an inflammatory cytokine elevated in response to radiation exposure, exerts pleiotropic effects by reinforcing immune suppression, stimulating angiogenesis, and sustaining a niche conducive to metastatic outgrowth [25,27]. The interplay between these factors establishes a permissive microenvironment that primes the lung for colonization by disseminated tumor cells [29].

Our findings align with previous reports indicating that low-dose radiation can modulate immune responses and promote angiogenesis [8,9]. However, our study expands upon these observations by identifying a mechanistic axis linking SDIR exposure to neutrophil-driven PMN remodeling and metabolic adaptations. Notably, SDIR-induced neutrophil accumulation was entirely abrogated by Bv8 blockade, which also prevented the associated upregulation of MMP9 and S100A8, confirming the pivotal role of Bv8 in mediating these effects.

Importantly, while SDIRs significantly accelerated PMN formation at early time points, differences between irradiated and non-irradiated conditions for MMP9 and S100A8 were no longer observed by day 14, as their expression levels increased equally in both experimental groups. This suggests that SDIR functions primarily as an early catalyst of niche priming. This is further supported by our observation that the Bv8 blockade, despite effectively preventing SDIR-induced neutrophil recruitment, did not abrogate the increase in TMV observed at later time points. These findings indicate that SDIR-driven metastasis involves mechanisms extending beyond neutrophil accumulation, potentially encompassing stromal activation, metabolic reprogramming, and vascular remodeling. This prompted us to conduct a proteomic analysis, which revealed profound metabolic reprogramming within the PMN as early as day 11 following SDIR exposure.

In agreement with previous studies, the PMN undergoes significant metabolic reprogramming, which further facilitates tumor cell colonization and outgrowth [29]. Increasing evidence suggests a shift from oxidative phosphorylation (OXPHOS) toward glycolysis and lactate production, a change that is essential for creating a permissive environment for metastasis [11]. A key driver of this metabolic shift is the activation of inducible nitric oxide synthase (NOS2) in response to inflammatory signals [11]. Nitric oxide inhibits key components of the mitochondrial electron transport chain, notably complex III (ubiquinol-cytochrome c reductase) and complex IV (cytochrome c oxidase), impairing mitochondrial respiration and further promoting a glycolytic phenotype [11].

This metabolic reprogramming leads to lactate accumulation, resulting in microenvironment acidification and immune suppression, both of which are hallmarks of a metastasis-permissive niche [30]. Our study contributes novel insights by demonstrating that SDIR exposure induces a significant downregulation of OXPHOS-related genes within the PMN. Proteome-wide GSEA revealed a significant depletion of the oxidative phosphorylation pathway (KEGG), with key components of the electron transport chain exhibiting reduced expression. This includes NDUFS4, NDUFV1, and NDUFS5, which are crucial for NADH oxidation and electron transfer in complex I, and UQCRC2, a core component of complex III involved in maintaining the proton gradient [33]. Additionally, the downregulation of NDUFA4, which regulates cytochrome c oxidase activity in complex IV, suggests further inhibition of mitochondrial electron flow [33]. Although we observed a reduction in ATP5PB and ATP5MG, key components of ATP synthase [33], the downregulation of critical subunits in complexes I, III, and IV appears to be the primary driver of OXPHOS disruption. Given that ATP synthase function relies on the proton gradient established by upstream complexes, we propose that its downregulation is a downstream effect of impaired electron transport, rather than a direct cause of the metabolic reprogramming in the PMN following SDIR exposure. Furthermore, GSEA in this study pointed to a marked decrease in the expression of PDHA1 and PDHB, core subunits of the pyruvate dehydrogenase complex, specifically driven by SDIR exposure. These enzymes are essential for converting pyruvate to acetyl-CoA, a critical step for entry into the tricarboxylic acid (TCA) cycle, thereby further promoting the metabolic shift away from mitochondrial respiration [32]. The accumulation of pyruvate in the cytoplasm is expected to drive its conversion to lactate, a process that would likely contribute to extracellular acidification, immune evasion, and stromal remodeling, all of which are key features that support metastatic progression [30]. In addition to these metabolic alterations, a significant decrease in fatty acid metabolism pathways (KEGG) is observed in the SDIR-exposed PMN. The downregulation of ACSL4 and ACSL5, enzymes responsible for the activation of long-chain fatty acids for mitochondrial oxidation [35], indicates a reduced capacity for fatty acid utilization. This alteration is further supported by a decrease in the expression of ECHS1 and HADHB, essential enzymes in the β-oxidation pathway, suggesting a reduction in fatty acid oxidation [35]. These changes indicate a shift away from fatty acid metabolism, potentially driving increased reliance on glycolysis and facilitating lipid accumulation within the PMN. This shift contributes to ECM remodeling and immune modulation, fostering a microenvironment that is conducive to tumor progression [30]. Our results also highlight the role of neutrophils in this altered metabolic landscape. Previous studies have established that neutrophils within the lung PMN accumulate neutral lipids, a process intricately regulated by lung-resident mesenchymal cells [36]. This lipid sequestration is driven by the suppression of adipose triglyceride lipase (ATGL), a key enzyme in triglyceride hydrolysis, leading to intracellular lipid accumulation [36]. Rather than serving as inert reservoirs, these lipid-laden neutrophils function as metabolic intermediaries, transferring stored lipids to metastatic tumor cells. This exchange fuels tumor cell bioenergetics, enhancing their survival, proliferation, and invasive potential. These findings underscore a broader metabolic crosstalk between immune and stromal components within the PMN, wherein neutrophils not only shape the inflammatory landscape but also actively reprogram the metabolic architecture of the metastatic niche [36].

SDIR exposure drives profound metabolic reprogramming within the PMN, characterized by the suppression of both OXPHOS and fatty acid oxidation, alongside an increased dependence on glycolysis. The downregulation of key enzymes in fatty acid metabolism, coupled with lipid accumulation, further highlights the metabolic shift that supports tumor progression [37]. Additionally, the accumulation of pyruvate and the consequent rise in lactate levels are expected to drive extracellular acidification, contributing to immune modulation [38]. This rewiring promotes a pro-inflammatory phenotype in neutrophils and macrophages while fostering an immunosuppressive environment, a hallmark of metastatic niches [29]. In parallel, the acidified environment is likely to activate fibroblasts and other stromal cells, inducing the secretion of matrix remodeling factors that facilitate tumor cell invasion [29]. Metastatic progression is shaped not only by the intrinsic properties of circulating tumor cells but also by the permissiveness of the PMN. Our findings reveal, for the first time, that the SDIR profoundly reprograms the metabolic landscape of the lung PMN, establishing conditions that facilitate tumor colonization. This highlights the need to integrate organ-specific radiation exposure and dose ranges into predictive models of metastasis, particularly in tumors with defined organotropism. By identifying metabolic pathways and immune-stromal interactions that drive SDIR-induced PMN remodeling, this study uncovers actionable targets for intervention. While the current findings offer novel insights into the impact of SDIRs on pre-metastatic niche formation, certain considerations should be kept in mind when interpreting the data. The use of a single animal model, though well-characterized, may not capture the full spectrum of human physiological responses to low-dose radiation. Another important limitation of this study is the absence of long-term follow-up after SDIR exposure. While our data reveal early and intermediate effects on PMN remodeling, they do not capture the potential delayed consequences of SDIRs, which could manifest beyond the timeframe of our current experimental design. Nonetheless, as cancer survivorship improves and the cumulative impact of radiation exposure becomes more clinically relevant, future research should explore the temporal evolution of SDIR-induced changes over longer durations. Nonetheless, these results provide a mechanistic foundation that opens important avenues for future translational research. Validating these pathways in patient samples or organotypic models and integrating them into studies of clinical radiotherapy will be essential to assess their broader applicability. In this light, our study should be viewed as a step toward a more integrated understanding of radiation-induced metastatic risk, with future work needed to bridge the gap to clinical implementation. These findings pave the way for precision oncology strategies aimed at mitigating radiation-induced metastatic risk. These insights have direct implications for optimizing radiotherapy protocols, not only in terms of dose and field design but also with regard to therapeutic timing. For instance, thoracic radiation fields might be adjusted to minimize low-dose spillover to distant, organ-specific metastatic sites, particularly in cancers with well-characterized organotropism. Future strategies should leverage these findings to refine patient stratification, adjust radiation plans accordingly, and explore the use of metabolic or immunomodulatory agents to counteract SDIR-driven niche formation. Together, these approaches can help balance local tumor control with long-term metastatic risk, supporting a more holistic model of precision radiotherapy.

## 4. Materials and Methods

### 4.1. Cell Culture

The 4T1 breast carcinoma cells (American Type Culture Collection, RRID:CVCL_0125) were maintained in Dulbecco’s modified Eagle’s medium GlutaMAX (DMEM, Gibco, Thermo Fisher Scientific, Waltham, MA, USA) with 10% (*v*/*v*) FBS (Gibco, Thermo Fisher Scientific, Waltham, MA, USA) and 1% (*v*/*v*) penicillin/streptomycin (Gibco, Thermo Fisher Scientific, Waltham, MA, USA). Cells were used at passage 5 and confirmed mycoplasma-free.

### 4.2. Animal Procedures

Animals were housed in individually ventilated cages (IVCs), with a maximum of five mice per cage, using corn cob bedding. Housing conditions were maintained within standard parameters: temperature at 22 ± 2 °C, relative humidity at 55 ± 10%, and a 14/12 h light/dark cycle. Animals had *ad libitum* access to standard laboratory rodent chow and filtered water. Environmental enrichment was routinely provided and included nesting material and cardboard tunnels. Animal health and welfare were monitored daily by trained and competent personnel, and any signs of distress or illness were promptly addressed, ensuring compliance with institutional and European Union regulations on animal care and use. Eight-week-old female Balb/c mice (Charles River Laboratories, Barcelona, Spain, RRID:IMSR_JAX:001026) were anesthetized with intraperitoneal ketamine-medetomidine (75 mg/kg and 1 mg/kg BW). Adequate sedation was confirmed by the absence of the paw withdrawal reflex, and anesthesia was reversed with atipamezole (5 mg/kg BW). Mice were shaved in the abdominal region and anesthetized before 4T1 cell injection and radiotherapy planning. To induce tumors, 4T1 cells (5 × 10^4^) were injected subcutaneously into the fourth right mammary gland in a 50 µL Matrigel/PBS (1:5) mixture. Mice received thoracic irradiation (0.3 Gy) on days 8–10 post-injection. At the experimental endpoint (days 11 or 14), lungs were collected for analysis by flow cytometry, ELISA, or qRT-PCR. For antibody treatment, Balb/c mice received intraperitoneal injections of anti-Bv8 monoclonal antibody (Genentech, Inc., South San Francisco, CA, USA) at 5 mg/kg, twice weekly for up to two weeks after 4T1 cell injection. Animals were randomly assigned to experimental groups using simple randomization, and all analyses were performed in a blinded manner to reduce bias. Sample size was determined by power analysis, assuming a significance level of 0.05 and a power of 0.9. Two animals were excluded from the final analysis on day 18 after tumor cell injection due to primary tumor weights below 0.05 g. Euthanasia was performed by cervical dislocation.

### 4.3. Irradiation Procedure

The setup for 3D reconstruction of the mice for treatment planning was as follows: the mice were positioned in an acrylic phantom to ensure proper thickness and enhance dose homogeneity during radiation therapy. A computed tomography (CT) scan was performed using a Siemens Somatom Sensation scanner, acquiring volumetric images reconstructed into 1 mm axial slices. These cross-sectional images were then transferred to the 3D computerized treatment planning system (TPS), Monaco (Elekta), for contouring the planning target volume (PTV), which encompassed the thoracic region of the mice. The radiotherapy treatment plan was designed within the same TPS, employing an isocentric dose distribution from two opposing fields (0° and 180°) with a 6 MV photon beam. The dose distribution was normalized to a reference point within the PTV. The dose calculation was performed using the clinical Monte Carlo algorithm, XVMC, for photons. A prescribed dose variation of 0.3 Gy was applied with a tolerance of ±10% within the PTV to ensure optimal dose coverage. Radiation treatment was administered at room temperature using a linear accelerator (Synergy, Elekta, Stockholm, Sweden) generating a 6 MV photon beam at a dose rate of 600 MU/min. To verify that the calculated dose matched the delivered dose to the PTV, regular quality control tests were conducted on the linear accelerator. These tests confirmed an uncertainty of less than 3% (k = 1) between the calculated and delivered doses. The absorbed dose to water for the radiation beam was determined following the IAEA TRS-398 code of practice. A total dose of 0.3 Gy was delivered to the thoracic region of the mice for three consecutive days. Control mice were sham irradiated (0.0 Gy) following the same procedures as the irradiated experimental groups. During the treatment protocol, the mice were anesthetized as previously described.

### 4.4. Protein Extraction and Digestion

Lung tissue proteins were extracted and solubilized with 100 mM Tris pH 8.5, 1% sodium deoxycholate, 10 mM tris(2-carboxyethyl)phosphine (TCEP), 40 mM of chloroacetamide and protease inhibitors for 10 min, at 95 °C and 100× *g*. Protein extract was sonicated for 10 cycles, 30 s on/30 s off (Bioruptor Plus, Diagenode, Seraing, Belgium). After centrifugation at 16.560× *g*, for 5 min, 100 μg of protein was processed for proteomic analysis following the solid-phase-enhanced sample-preparation (SP3) protocol [39]. Enzymatic digestion was performed with Trypsin/LysC (2 μg), overnight at 37 °C and 100× *g*. The resulting peptides were cleaned up and desalted with C18 micro-columns and further quantified.

### 4.5. NanoLC-MS/MS

Protein identification was performed by nanoLC-MS/MS. This equipment is composed of an Ultimate 3000 liquid chromatography system coupled to a Q-Exactive Hybrid Quadrupole-Orbitrap mass spectrometer (Thermo Fisher Scientific, Waltham, MA, USA). In total, 500 ng of peptides were loaded onto a trapping cartridge (Acclaim PepMap C18 100 Å, 5 mm × 250 µm i.d., 160454, Thermo Fisher Scientific, Waltham, MA, USA) in a mobile phase of 2% ACN, 0.1% FA at 10 µL/min. After 3 min loading, the trap column was switched in-line to a 50 cm by 75 μm inner diameter EASY-Spray column (ES803, PepMap RSLC, C18, 2 μm, Thermo Fisher Scientific, Waltham, MA, USA) at 300 nL/min. Separation was generated by mixing A: 0.1% FA and B: 80% ACN, with the following gradient: 5 min (2.5% B to 10% B), 120 min (10% B to 30% B), 20 min (30% B to 50% B), 5 min (50% B to 99% B), and 10 min (hold 99% B). Subsequently, the column was equilibrated with 2.5% B for 17 min. Data acquisition was controlled by Tune 2.9 software and Xcalibur 4.0 (Thermo Fisher Scientific, Waltham, MA, USA, RRID:SCR_014593).

The mass spectrometer was operated in data-dependent (dd) positive acquisition mode alternating between a full scan (*m*/*z* 380–1580) and subsequent HCD MS/MS of the 10 most intense peaks from full scan (normalized collision energy of 27%). ESI spray voltage was 1.9 kV. Global settings: Lock masses best (*m*/*z* 445.12003), lock mass injection Full MS, chrom. Peak width (FWHM) of 15 s. Full scan settings: Resolution of 70 k (*m*/*z* 200), AGC target 3 × 10^6^, and maximum injection time of 120 ms. dd settings: Minimum AGC target 8 × 10^3^, intensity threshold of 7.3 × 10^4^, charge exclusion: unassigned, 1, 8, >8, peptide match preferred, exclude isotopes on, and dynamic exclusion 45 s. MS2 settings: Microscans 1, resolution of 35 k (*m*/*z* 200), AGC target 2 × 10^5^, maximum injection time of 110 ms, isolation window 2.0 *m*/*z*, isolation offset 0.0 *m*/*z*, and spectrum data type profile.

### 4.6. Protein Identification and Quantification

Protein identification and quantitative analysis were performed by Proteome Discoverer™ (Thermo Fisher Scientific, Waltham, MA, USA), MaxQuant Version 1.5.3.30 and VEMS considering the information from the UniProt protein database (RRID:SCR_002380) for *Mus musculus* taxonomic selection. Quantitative analysis was compared and merged to create a consensus quantitative report to minimize computational artifacts. Quantitation was performed on the peptide and protein level based on iBAQ ion counts.

### 4.7. Database-Dependent Search of MS Data

Mass accuracy was set to 5 ppm on the peptide level and 0.01 Da on the fragment ions. A maximum of four missed cleavages was used. Carbamidomethyl (C) was set as a fixed modification. Methionine oxidation and N-terminal protein acetylation were set as variable modifications. The MS/MS data was searched against all reviewed mouse proteins from UniProt with the concatenation of all the sequences in reverse, maintaining only lysine and arginine in place. The data was searched and quantified with both MaxQuant Version 1.5.3.30 and VEMS [40]. For MaxQuant, the variable modifications such as methionine oxidation, N-terminal protein acetylation, tyrosine phosphorylation, threonine phosphorylation, and serine phosphorylation were included.

### 4.8. Quantitative Analysis of MS Data

Quantitative data from MaxQuant and VEMS were analyzed using the R statistical programming language. Protein iBAQ values from the two programs were preprocessed by removing common MS contaminants, followed by Log_2_(x + 1) transformation and quantile normalization. The distribution of quantitative values in each sample for each processing method was analyzed using boxplots in R. To identify differentially expressed proteins, R (v4.4.0) and the limma package (v3.60.4; RRID:SCR_010943) were used to compare each tumor-bearing experimental group with one another, as well as with the control group. Multiple testing correction was applied using the Benjamini-Hochberg method to control the false discovery rate (FDR). Proteins with an FDR < 0.1 were considered differentially expressed. No fold-change cutoff was applied.

### 4.9. Functional Enrichment Analysis

GSEA was performed to identify functionally enriched pathways among differentially expressed proteins. Enrichment analysis was carried out using KEGG pathway annotations (RRID:SCR_012773), considering the differential expression of proteins between experimental groups. *p*-values were estimated by comparing the empirical enrichment score (ES) of a gene set relative to a null distribution of ESs derived from permuting the gene set 1000 times and then adjusting for multiple hypothesis testing.

Proteins assigned to significantly enriched pathways were extracted and categorized based on functional annotations. The top enriched pathways were ranked according to statistical significance, and results were visualized as bar plots representing normalized enrichment scores. All data processing and statistical analysis were conducted using R statistical programming language.

### 4.10. Circulating Cytokine Quantification

Lungs were perfused with PBS and collected in tubes containing lysing matrix D (MP Biomedicals, Aurora, OH, USA) and 0.5 mL of PBS supplemented with an EDTA-free Protease Inhibitor Cocktail (Roche, Basel, Switzerland). Lung tissue was homogenized twice using a Mini-Beadbeater for 45 s. Homogenates were centrifuged at 16.060× *g* for 10 min, and the supernatant was collected. Matrix metallopeptidase 9 (MMP9), S100 calcium-binding protein A8 (S100A8), S100 calcium-binding protein A9 (S100A9), granulocyte-colony stimulating factor (GCSF), and fibronectin levels were quantified in the supernatant using ELISA kits (Abcam, Cambridge, UK), following the manufacturer’s instructions.

### 4.11. Tissue Digestion and Flow Cytometry

Pulmonary cells were isolated from well-perfused lungs. Lung tissue was finely chopped and digested with Type IV collagenase (0.5 mg/mL; Sigma-Aldrich, St. Louis, MO, USA) and DNase I (10 µg/mL; Sigma-Aldrich, St. Louis, MO, USA) for 30 min at 37 °C with agitation (100× *g*). The resulting cell suspension was filtered through a 70 µm nylon cell strainer (Falcon Corning Incorporated, Corning, NY, USA), and erythrocytes were lysed osmotically using RBC Lysis Buffer (BioLegend, San Diego, CA, USA) for 5 min in the dark. Cells were then counted.

For surface staining, cells were Fc-blocked with mouse anti-CD16/32 (Thermo Fisher Scientific, Waltham, MA, USA, Cat# 14-0161-82, RRID:AB_467133), followed by incubation with an antibody mix for 1 h at room temperature in the dark. The following monoclonal antibodies were used: anti-CD45 (BD Biosciences, San Jose, CA, USA, Cat# 559864, RRID:AB_398672), anti-CD3ε (Thermo Fisher Scientific, Waltham, MA, USA, Cat# 45-0031-82, RRID:AB_1107000), anti-CD11b (BD Biosciences, San Jose, CA, USA, Cat# 563168, RRID:AB_2716860), anti-F4/80 (Invitrogen, Carlsbad, CA, USA, Cat# 25-4801-82, RRID:AB_469653), and anti-Ly6C (Invitrogen, Carlsbad, CA, USA, Cat# 53-5932-82, RRID:AB_2574427). Zombie Aqua Dye (BioLegend, San Diego, CA, USA, Cat# 423101) as added to the final suspension for 30 min at room temperature to stain and exclude dead cells. Samples were acquired using a FACS Fortessa™ (BD Biosciences, San Jose, CA, USA), and data were analyzed with FlowJo™ v10 software (Tree Star, Inc., Ashland, OR, USA, RRID:SCR_008520).

### 4.12. RNA Extraction, cDNA Synthesis, and Quantitative Real-Time PCR

Total RNA was extracted using an RNeasy^®^ Micro Kit (QIAGEN, Hilden, Germany) according to the instructions of the manufacturer, for animal tissue. For the synthesis and pre-amplification of cDNA, the RT2 Nano PreAMP cDNA synthesis kit (QIAGEN, Hilden, Germany) was used with two rounds of pre-amplification, using the same genes described below. The mRNA expression levels were assessed by qRT-PCR using a Power SYBR^®^ Green system (Invitrogen, Carlsbad, CA, USA) on a 7500/7500 Fast Real-Time PCR System (Applied Biosystems, Foster City, CA, USA). The housekeeping gene was 18S. The initial denaturation step was 10 min at 95 °C, followed by 50 cycles of 15 s at 95 °C, and 1 min at 60 °C. The relative quantification was performed through the 2^−ΔCt^ method, where ΔCt = Ct (sample) − Ct (reference gene). Data for each animal are represented as a Log2 of fold change (2^−ΔCt^), relative to the fold change average of the 0.0 Gy group. Gene-specific primers are detailed as follows (5′–3′):

*Bv8* Fw: GAAGGGCGAAGAGAAGAAAGAG
*Bv8* Rev: TCCGGGCCAAGCAAATAA
*Il6* Fw: GTCTGTAGCTCATTCTGCTCTG
*Il6* Rev: GAAGGCAACTGGATGGAAGT
*Tnf* Fw: CTACCTTGTTGCCTCCTCTTT
*Tnf* Rev: GAGCAGAGGTTCAGTGATGTAG

### 4.13. Stereology Analysis

Lungs were formalin-fixed, paraffin-embedded, and sectioned at a uniform interval (T) of 200 µm with a random start, yielding a total of eight sections per lung. All sections were stained with H&E. Volume estimates were obtained using the Cavalieri principle [41]: V = T × a/p × ∑Pi, where T is the thickness of the slabs, Pi is the number of points hitting the structure of interest, and a/p is the area associated with each point. For point counting, step lengths in both the x- and y-directions were fixed, enabling the evaluation of 20% of the total area using a 10x objective. The meander sampling resulted in approximately 30 fields evaluated per section. Quantification was performed by overlaying a point grid onto 10× fields of view and counting the number of points hitting the lung and those hitting lung metastases. A 5 × 5 (25-point) grid was used. Each point has an associated area (a/p), which is multiplied by the number of points counted. The circled point in the upper left-hand corner was counted each time it hit the lung (A), with an associated area 25 times larger than the individual points counted when hitting metastases (B). The area fraction of lung metastases, representing the percentage of lung occupied by metastases, was calculated by dividing the number of points hitting metastases by the number hitting the lung. Digital slides were acquired using the Hamamatsu NanoZoomer slide scanner, and measurements were performed with Visiopharm stereology software (version 2019, Visiopharm A/S, Hørsholm, Denmark). Stereology was conducted at the Histopathology Unit at IGC.

### 4.14. Statistics

For multiple-group comparisons, one-way ANOVA with Bonferroni post hoc correction was used for normally distributed data. When variance was unequal, Welch’s ANOVA with Tamhane’s T2 post hoc test was applied. For non-normally distributed data, the Kruskal–Wallis test with Dunn’s multiple comparison adjustment was used.

For two-group comparisons, an independent two-tailed unpaired *t*-test was performed for normally distributed data, with Welch’s correction applied in cases of unequal variance. Assumptions of normality and homogeneity of variances were tested using the Shapiro-Wilk and Levene’s tests, respectively. Statistical analyses were performed using GraphPad Prism 8 (Graphpad Software, LLC Company Profile, San Diego, CA, USA, RRID:SCR_002798). All *p*-values are two-sided, with *p* < 0.05 considered statistically significant. Sample sizes (n) per experiment are provided in the corresponding figure legends.

## Data Availability

Raw proteomics data have been deposited in the ProteomeXchange Consortium via the PRIDE partner repository with the dataset identifier PXD062108.

## Supplementary Materials

The following supporting information can be downloaded at: https://www.mdpi.com/article/10.3390/ijms26136145/s1.
