# Supplementary material for: Subtherapeutic Dose of Ionizing Radiation Reprograms the Pre-Metastatic Lung Niche, Accelerating Its Formation and Promoting Metastasis
Source: Int J Mol Sci. 2025 Jun 26;26(13):6145. doi: 10.3390/ijms26136145 (PMC12249717; doi:10.3390/ijms26136145)
Supplement: Supplementary file 1 [file ijms-26-06145-s001.zip › ijms-3671918-supplementary.pdf]

SUPPLEMENTARY MATERIALS

Subtherapeutic-Dose of Ionizing Radiation Reprograms the Pre-Metastatic Lung Niche, Accelerating Its Formation and Promoting Metastasis

Paula de Oliveira [1], Inês Sofia Vala [1], Pedro Faísca[2], Joao C Guimaraes [3], Filomena Pina [4], Esmeralda Poli [4], Isabel Diegues [4], Hugo Osório [5, 6, 7], Rune Matthiesen [8], Karine Serre [9], Susana Constantino Rosa Santos [1,*]

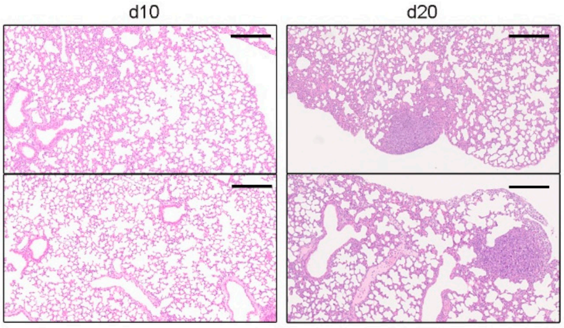

Figure S1. Histological analysis of lung metastasis development following primary tumor induction in 4T1 breast cancer-bearing mice. Lung tissue samples were collected from BALB/c mice injected with 4T1 breast cancer cells on day 0. Representative haematoxylin and eosin-stained histological sections from day 10 (d10) and day 20 (d20) are shown. Scale bars: 250 µm.

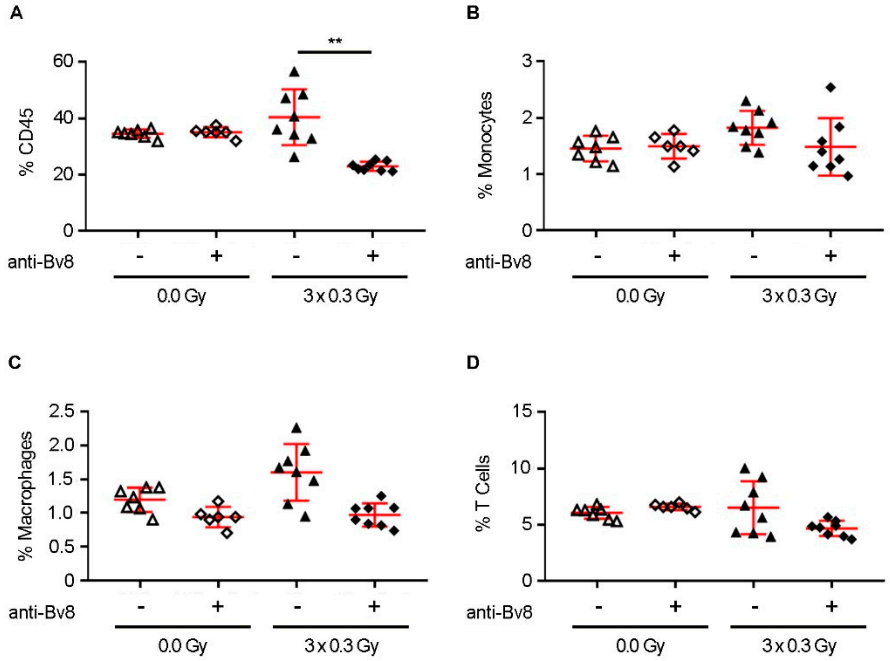

Figure S2. Effect of anti-Bv8 treatment and LDIR on immune cell recruitment in lung PMN of 4T1 tumor-bearing mice. Lung tissue samples from BALB/c mice injected with 4T1 breast cancer, sham-irradiated (0.0 Gy) or exposed to 0.3 Gy for 3 consecutive days (3×0.3 Gy), with or without anti-Bv8 treatment, were collected on day 11. The percentage of (**A**) total $CD45^+$ (**B**) monocytes ($CD45^+CD11b^+Ly6C^+F4/80int$) (**C**) macrophages ($CD45^+CD11b^+F4/80^+$) and (**D**) T cells ($CD45^+CD11b^-CD3^+$) was quantified by flow cytometry. (**A-D**) Individual data and mean ± SD (in red) are shown; n=6-8. Between-group changes were assessed using one-way ANOVA followed by Bonferroni post-hoc test, or Welch's ANOVA with Tamhane T2 post-hoc test when unequal variance was verified. Statistical significance: $P<0.01$ (**).

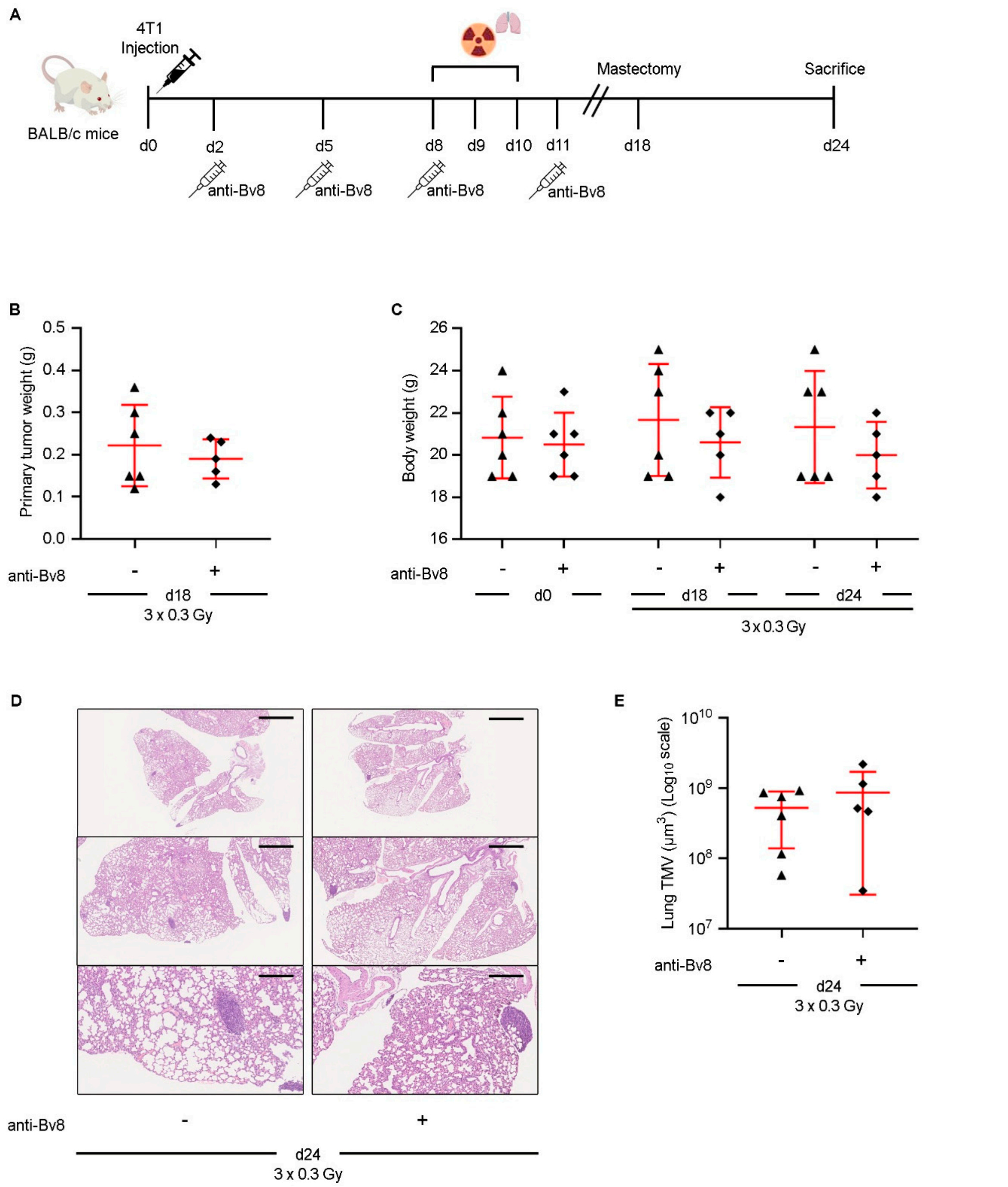

Figure S3. Anti-Bv8 treatment in lung metastasis development in irradiated 4T1 tumor-bearing mice. (**A**) Experimental design: BALB/c mice were subcutaneously injected with 4T1 tumor cells on day 0 (d0). Anti-Bv8 treatment was administered intraperitoneally on days 2, 5, 8 and 11 (d2, d5, d8, d11). Mice were exposed to 0.3 Gy for 3 consecutive days (3x0.3Gy) on days 8, 9 and 10 (d8-d10). Mastectomy was performed on day 18 (d18) and the experimental endpoint was reached on day 24 (d24). (**B**) Primary tumor weight was measured at d18. (**C**) Body weight was monitored on d0, d18, and d24. (**D**) Representative haematoxylin and eosin-stained histological sections are shown. Scale bars: 2.5 mm (upper panels), 1.0 mm (middle panels), 250 µm (lower panels). (**E**) Total metastatic volume (TMV) in lungs was quantified stereologically on d24. (**B-C**, **E**) Individual data and mean ± SD (in red) are shown; n=5-6. Normal distribution was assumed; an independent two-tailed *t*-test with Welch's correction for unequal variances was applied; no significant differences were found.

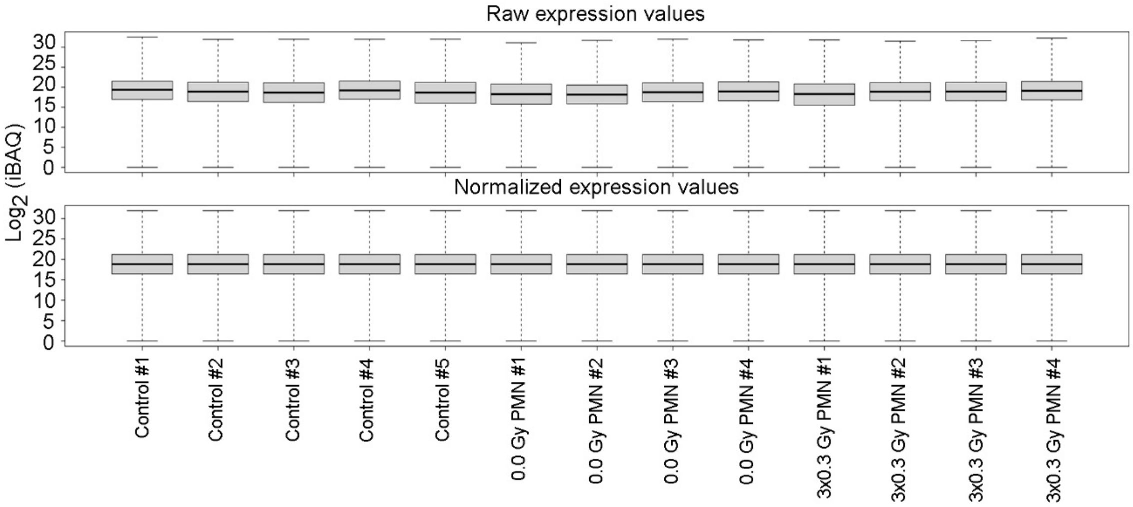

Figure S4. Quality control of proteomic data across experimental groups in lung tissue following 4T1 tumor cell injection**.** Lung tissue samples from control, 0.0 Gy PMN, and 3×0.3 Gy PMN groups were collected on day 11 post-4T1 tumor cell injection and analyzed by mass spectrometry. Boxplots represent the distribution of Log_2(iBAQ) expression values for each sample, before (top) and after (bottom) normalization. The overall distribution of quantitative data was comparable across all experimental conditions, confirming data consistency and reproducibility.

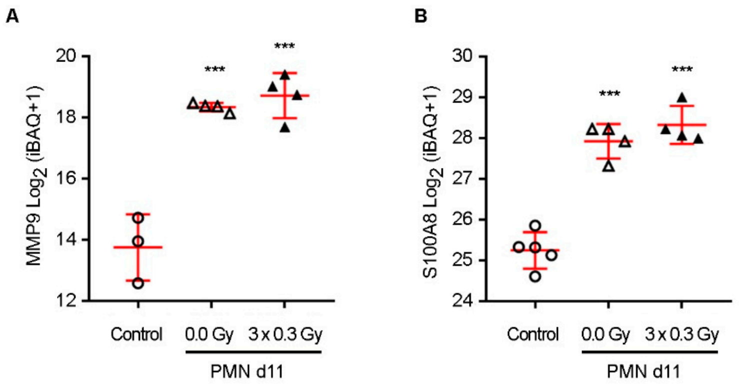

Figure S5. Proteomic analysis of lung PMN reveals increased MMP9 and S100A8. Lung tissue proteomes from control, 0.0 Gy PMN, and 3×0.3 Gy PMN groups were analyzed by mass spectrometry-based proteomics on day 11 post-4T1 tumor cell injection. Quantitative Log_2(iBAQ+1) expression values of A, MMP9 and B, S100A8 are shown. Individual data points and mean ± SD (in red) are displayed (n=4). Statistical significance was determined using the limma package in R. Statistical significance: $P<0.05$ (*); $P<0.01$ (**); $P<0.001$ (***).
